# Differences in network controllability and regional gene expression underlie hallucinations in Parkinson’s disease

**DOI:** 10.1093/brain/awaa270

**Published:** 2020-10-29

**Authors:** Angeliki Zarkali, Peter McColgan, Mina Ryten, Regina Reynolds, Louise-Ann Leyland, Andrew J Lees, Geraint Rees, Rimona S Weil

**Affiliations:** a1 Dementia Research Centre, University College London, 8-11 Queen Square, London, WC1N 3AR, UK; a2 Huntington’s Disease Centre, University College London, Russell Square House, London, WC1B 5EH, UK; a3 Department of Neurodegenerative Disease, UCL Institute of Neurology, 10-12 Russell Square House, London, UK; a4 Reta Lila Weston Institute of Neurological Studies, 1 Wakefield Street, London, WC1N 1PJ, UK; a5 Institute of Cognitive Neuroscience, University College London, 17-19 Queen Square, London, WC1N 3AR, UK; a6 Wellcome Centre for Human Neuroimaging, University College London, 12 Queen Square, London, WC1N 3AR, UK; a7 Movement Disorders Consortium, University College London, London WC1N 3BG, UK

**Keywords:** Parkinson’s disease, visual hallucinations, diffusion weighted imaging, regional gene expression, controllability

## Abstract

Visual hallucinations are common in Parkinson’s disease and are associated with poorer prognosis. Imaging studies show white matter loss and functional connectivity changes with Parkinson’s visual hallucinations, but the biological factors underlying selective vulnerability of affected parts of the brain network are unknown. Recent models for Parkinson’s disease hallucinations suggest they arise due to a shift in the relative effects of different networks. Understanding how structural connectivity affects the interplay between networks will provide important mechanistic insights. To address this, we investigated the structural connectivity changes that accompany visual hallucinations in Parkinson’s disease and the organizational and gene expression characteristics of the preferentially affected areas of the network. We performed diffusion-weighted imaging in 100 patients with Parkinson’s disease (81 without hallucinations, 19 with visual hallucinations) and 34 healthy age-matched controls. We used network-based statistics to identify changes in structural connectivity in Parkinson’s disease patients with hallucinations and performed an analysis of controllability, an emerging technique that allows quantification of the influence a brain region has across the rest of the network. Using these techniques, we identified a subnetwork of reduced connectivity in Parkinson’s disease hallucinations. We then used the Allen Institute for Brain Sciences human transcriptome atlas to identify regional gene expression patterns associated with affected areas of the network. Within this network, Parkinson’s disease patients with hallucinations showed reduced controllability (less influence over other brain regions), than Parkinson’s disease patients without hallucinations and controls. This subnetwork appears to be critical for overall brain integration, as even in controls, nodes with high controllability were more likely to be within the subnetwork. Gene expression analysis of gene modules related to the affected subnetwork revealed that down-weighted genes were most significantly enriched in genes related to mRNA and chromosome metabolic processes (with enrichment in oligodendrocytes) and upweighted genes to protein localization (with enrichment in neuronal cells). Our findings provide insights into how hallucinations are generated, with breakdown of a key structural subnetwork that exerts control across distributed brain regions. Expression of genes related to mRNA metabolism and membrane localization may be implicated, providing potential therapeutic targets.

## Teaser

Visual hallucinations in Parkinson’s disease are associated with connectivity loss, but the factors underlying selective regional vulnerability are unknown. Zarkali *et al.* show that hallucinations are associated with the breakdown of a structural subnetwork critical for brain integration, with distinct gene expression patterns.

## Introduction

Complex visual hallucinations are common in Parkinson’s disease, affecting 30–70% of patients (Fénelon *et al.*, 2000; Hely *et al.*, 2008). They are frequently distressing and distracting, and are a harbinger of dementia (Hobson and Meara, 2004; Galvin *et al.*, 2006). Furthermore, they are associated with increased mortality (Goetz and Stebbins, 1995), increased carer burden (Aarsland *et al.*, 2000) and worse quality of life (McKinlay *et al.*, 2007). They are also the strongest predictor of nursing home placement in patients with Parkinson’s disease (Aarsland *et al.*, 2000). Despite their impact, our understanding of how visual hallucinations are produced remains limited (Fénelon *et al.*, 2000; Weil *et al.*, 2016).

Recent models for Parkinson’s disease hallucinations suggest they arise due to a shift in the relative effects of different networks, or a failure to integrate sensory input and prior knowledge during visual perception (Muller *et al.*, 2014). Indeed, there is evidence for both impaired sensory accumulation (O’Callaghan *et al.*, 2017) and over-reliance on prior knowledge in Parkinson’s disease hallucinations (Zarkali *et al.*, 2019). Aberrant default mode network (DMN) activation is seen in patients with Parkinson’s disease and hallucinations (Yao *et al.*, 2014). A useful recent model is that visual hallucinations arise due to breakdown in connectivity of networks involved in attention and conscious perception, with overactivity of the DMN and failure to engage the dorsal attention network (Shine *et al.*, 2014, 2015; Onofrj *et al.*, 2017, 2019).

We recently showed that white matter connectivity is decreased in the splenium of the corpus callosum and the left posterior thalamic radiation in Parkinson’s disease with visual hallucinations (PD-VH) (Zarkali *et al.*, 2020). Broadly reduced connectivity strength has also been reported, preferentially affecting nodes of the ‘diverse club’, areas that are proposed to integrate across more specialist modules (Hall *et al.*, 2019). However, these studies do not directly examine the impact that structural connectivity changes have on functional dynamics and cannot address the factors that make specific brain regions more vulnerable to white matter loss. 

Controllability is a powerful emerging analysis technique that combines structural connectivity measures and linear estimates of local dynamics to provide a metric of the extent of influence of one part of the network over other parts of the brain and in changing brain states (Gu *et al.*, 2015). Given the emphasis on shifts between brain networks as a key driver of Parkinson’s disease hallucinations (Muller *et al.*, 2014), brain controllability is likely to provide important insights into how hallucinations arise in Parkinson’s disease.

The underlying biological processes that determine vulnerability of specific brain regions in Parkinson’s disease hallucinations remain unclear but differences in regional gene expression are likely to contribute. Regional gene expression in health has been shown to predict white matter connectivity loss in Huntington’s disease (McColgan *et al.*, 2018) and schizophrenia (Romme *et al.*, 2017) and expression of candidate genes has been associated with cortical atrophy in Parkinson’s disease (Freeze *et al.*, 2018, 2019). Characterizing potential changes in regional gene expression linked to connectivity loss in Parkinson’s disease hallucinations may provide important insights into the underlying biological processes that drive the interplay between networks.

Here, we aimed to clarify the structural connectivity changes in patients with Parkinson’s disease visual hallucinations (PD-VH) and shed light on the pathological processes that drive connectivity loss. To this end, we: (i) used network-based statistics to test whether structural connectivity is reduced in PD-VH; (ii) performed controllability analysis at whole-network and subnetwork level to assess the effect structural changes have on whole brain function. We hypothesized that areas which usually exert large control over the rest of the brain will be preferentially affected in PD-VH; (iii) used gene expression data from the Allen Human Brain Atlas (AHBA) to identify whether differences in regional gene expression could explain vulnerability of specific brain regions to connectivity loss in PD-VH; and (iv) performed enrichment analysis on the identified gene expression patterns associated with connectivity loss in PD-VH to clarify the biological and cell processes driving this connectivity loss.

## Materials and methods

### Participants

We recruited 100 patients with Parkinson’s disease to our UK centre from affiliated clinics and 34 unaffected controls from spouses as well as volunteer databases. All consecutive participants that were referred and were eligible for the study were recruited (no history of traumatic brain injury or other major psychiatric or neurological disorder and no contraindication to MRI and diagnosis within 10 years for Parkinson’s disease participants). Patients with Parkinson’s disease satisfied the Queen Square Brain Bank Criteria for Parkinson’s disease (Daniel and Lees, 1993). The study was approved by our ethics committee and participants provided written informed consent.

Patients with Parkinson’s disease were classified as hallucinators (PD-VH) if they scored ≥1 on Item 1.2 of the Movement Disorder Society Unified Parkinson’s Disease Rating Scale (MDS-UPDRS) [‘Over the past week have you seen, heard, smelled or felt things that were not really there?’ (Goetz *et al.*, 2008)]. Further information on hallucinatory experiences was collected using the University of Miami Hallucinations Questionnaire (Papapetropoulos *et al.*, 2008). Nineteen patients with Parkinson’s disease scored ≥1 and were classified as PD-VH, whilst 81 patients did not and were classified as non-hallucinators (PD-non-VH). None of the non-hallucinators had a history of previous hallucinations.

Participants underwent a series of clinical assessments. The MDS-UPDRS part III was used to assess motor function (Goetz *et al.*, 2008). The Mini-Mental State Examination (MMSE) and Montreal Cognitive Assessment (MoCA) were used as measures of general cognition (Dalrymple-Alford *et al.*, 2010; Creavin *et al.*, 2016). LogMAR was used to assess visual acuity (Sloan, 1959). The D15 was used to assess colour vision (Farnsworth, 1947) and the Pelli-Robson test to assess contrast sensitivity (Pelli *et al.*, 1988). Sniffin’ Sticks were used to assess smell (Hummel *et al.*, 1997). The Hospital Anxiety and Depression Scale (HADS) was used to assess mood (Zigmond and Snaith, 1983) and the REM Sleep Behaviour Disorder Questionnaire (RBDSQ) to assess sleep (Stiasny-Kolster *et al.*, 2007). Levodopa dose equivalence scores (LEDD) were calculated for Parkinson’s disease participants using the conversion described by Tomlinson *et al.* (2010).

### Structural connectivity data

#### Data acquisition

All MRI data were acquired on a 3 T Siemens Magnetom Prisma scanner (Siemens) with a 64-channel head coil. Diffusion-weighted imaging (DWI) was acquired with the following parameters: b = 50 s/mm^2^/17 directions, b = 300 s/mm^2^/8 directions, b = 1000 s/mm^2^/64 directions, b = 2000 s/mm^2^/64 directions, 2 × 2 × 2 mm isotropic voxels, echo time = 3260 ms, repetition time: 58 ms, 72 slices, 2 mm thickness, acceleration factor = 2. Acquisition time for DWI was ∼10 min. A 3D MPRAGE (magnetization prepared rapid acquisition gradient echo) image (voxel size 1 × 1 × 1 mm, echo time: 3.34 ms, repetition time: 2530 ms, flip angle = 7°) was also obtained and was used to compute intracranial volume using SPM12.

#### Data processing

**Figure 1:**
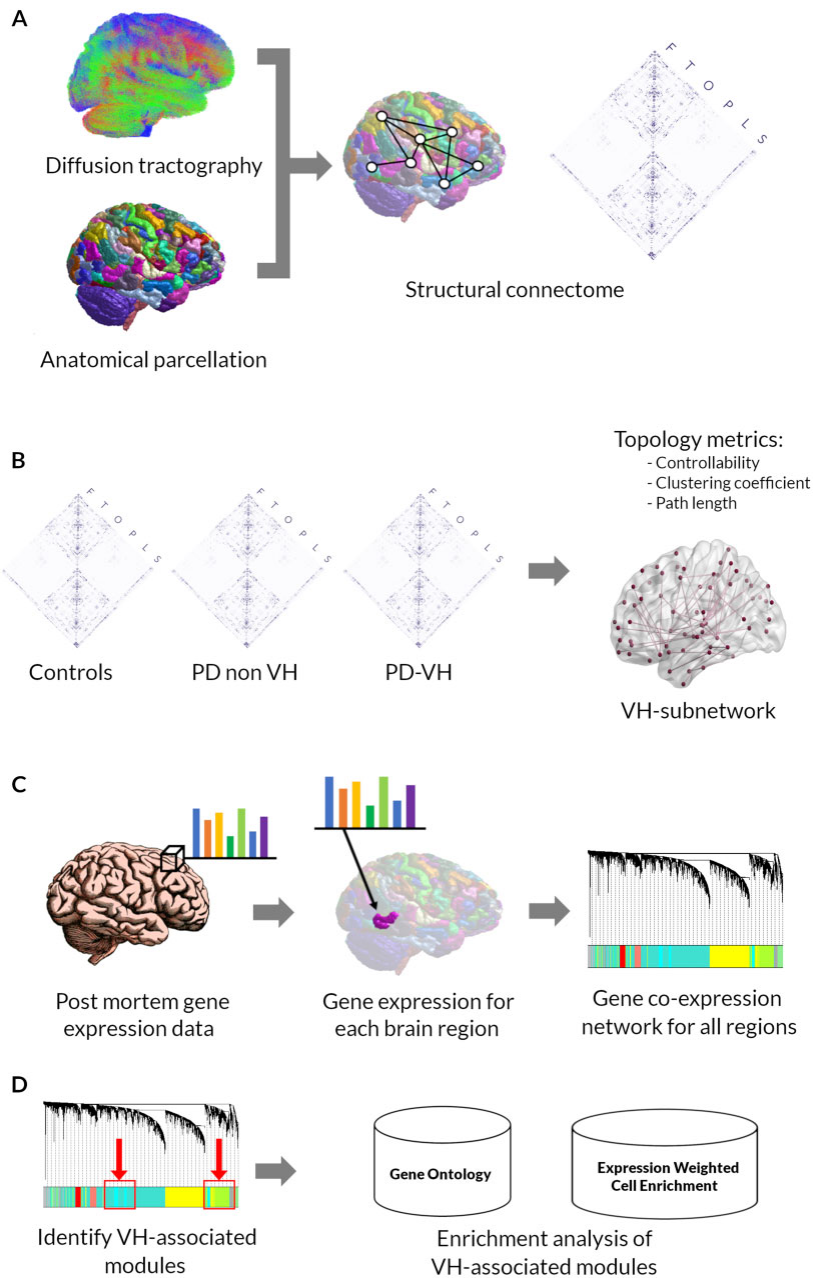
**Overview of the study methodology.** (**A**) Anatomically constrained tractography was used to determine white matter streamlines from diffusion weighted imaging data for each participant. Diffusion data were combined with an anatomical parcellation of 379 brain regions (360 cortical, 19 subcortical) using the Glasser atlas to generate a connectivity matrix for each participant. (**B**) Structural connectomes were compared between groups. First, global topology metrics (degree strength, path length, clustering coefficient) and controllability were calculated for each participant and compared between Parkinson’s disease (PD) and controls, and PD-VH and PD-non-VH. Second, network-based statistics was performed (contrasts of interest Parkinson’s disease versus controls and PD-VH versus PD-non-VH, age and total intracranial volume included as covariates) resulting to the identification of a VH-subnetwork of reduced connectivity strength. (**C**) Gene expression data were extracted from the AHBA and mapped into the 180 cortical regions from the left hemisphere according to our anatomical parcellation and an average regional gene expression was calculated for each gene for each cortical region. Gene co-expression network analysis was then performed for the 180 regions resulting to a network of 27 modules. (**D**) The modules of the resulting gene co-expression network were further examined to identify the modules associated with the VH subnetwork: the summary profile (eigengene) for each module was correlated with presence in the VH subnetwork. Two modules were significantly associated after correction for multiple comparisons, one down-weighted (cyan module) and one up-weighted (greenyellow module). Gene significance (the absolute value) of correlation between the gene and the trait (region’s presence in the VH subnetwork) was then calculated for each gene of the two VH-associated module. Enrichment analyses were then performed using the gene lists for these two modules, ranked by gene significance. F = frontal; L = limbic; O = occipital; P = parietal; S = subcortical; T = temporal.

An overview of the study methodology is seen in Fig. 1. Cortical regions of interest were generated by segmenting a T_1_-weighted image using the Glasser atlas in FreeSurfer (Glasser *et al.*, 2016) and subcortical regions of interest from the built-in Freesurfer parcellation (Fischl *et al.*, 2002). This resulted in 360 cortical regions (180 regions from each hemisphere) and 19 subcortical regions. The Glasser atlas was chosen as it is based on a large number of participants (210 healthy young adults), which were precisely aligned (Glasser *et al.*, 2016), and in a recent comparison between different parcellation methods, it showed good performance across the board when compared with other methods (Arslan *et al.*, 2018).

Diffusion-weighted images underwent denoising (Veraart *et al.*, 2016), removal of Gibbs ringing artefacts (Kellner *et al.*, 2016), eddy-current and motion correction (Anderson, 2006) and bias field correction (Tustison *et al.*, 2010). Diffusion tensor metrics were calculated and constrained spherical deconvolution (CSD) performed, as implemented in MRtrix (Hollander *et al.*, 2016). The raw T_1_-weighted images were registered to the diffusion weighted image using FLIRT (Greve and Fischl, 2009) and five-tissue anatomical segmentation was performed using the 5ttgen script in MRtrix. All resulting anatomical segmentations were visually inspected pre and post registration. Anatomically constrained tractography was then performed with 10 million streamlines (Smith *et al.*, 2012) using the iFOD2 tractography algorithm (Tournier *et al.*, 2010) with dynamic seeding, as implemented in MRtrix. To improve anatomically constrained tractography (ACT) performance we used the -backtrack option, which allows tracks to be truncated and retracked in case of poor termination and the -crop_at_gmwmi option which crops streamline end points more precisely as they cross the grey matter–white matter interface. We then applied the spherical deconvolution informed filtering of tractograms (SIFT2) algorithm (Smith *et al.*, 2015) to reduce biases. SIFT2 uses information from the fibre orientation distribution to determine a cross sectional area for each streamline and generate streamline volume estimates between regions whilst using the whole connectome (Smith *et al.*, 2015). The resulting set of streamlines was used to construct the structural brain network.

#### Structural connectome construction

For each participant, we generated a structural connectivity map by determining whether each pair of regions of interest were connected by a streamline; connections were weighted by streamline count and a cross-sectional area multiplier (Smith *et al.*, 2015). Then, connections were combined into 379 × 379 undirected and weighted connectivity matrices. In accordance with SIFT2 recommendations, we did not apply a threshold to connectivity matrices (Smith *et al.*, 2015) (Fig. 1A).

#### Network topology and controllability

Network control theory is an emerging analysis technique that provides mathematically-derived predictions for the impact of structural connectivity on brain function (Gu *et al.*, 2015; Betzel *et al.*, 2016). In contrast to graph theory metrics that describe the static organization of a network, network control theory models the role of a specific brain region in regulating whole-brain network function. The application of network control theory in brain dynamics has been previously detailed (Gu *et al.*, 2015). In brief, neural states can be mathematically described as simulated states (*x*) of network with *k* nodes over time steps *t* using the following equation:

(1) $$x_{t+1}\;=\;Ax_{t}+Bu_{t}$$

where *x_t_* is a vector of all simulated states of all nodes *k* at time *t*, *t* are discrete time steps (*t *=* *1, 2, …), *A_ke_* is the structural connectivity matrix for the network with *k* nodes and *e* edges, *B* is a matrix of the control nodes in the network and *u_t_* is the energy applied to the control nodes *B* at time *t*.

Subsequently, the influence of each region on brain function is quantified using the metric of controllability. Average controllability for a control node is a measure of the node’s ability to influence other nodes within the network, specifically to drive the network into different states. It is calculated as the average energy, or effort, required to reach all possible states of the system. Regions with high average controllability can therefore drive the brain network to many easily reachable states.

We assessed average controllability at node- and network-level in PD-VH, PD-non-VH and controls, using code available at: https://complexsystemsupenn.com/s/controllability_code-smb8.zip.

Finally, we calculated connectome density and global network metrics of segregation and integration using the Brain Connectivity Toolbox (Bullmore and Sporns, 2009). These included: clustering coefficient a metric of segregation, and characteristic path length, computed as the average of the shortest path length across all nodes (Fig. 1B).

### Mapping gene expression data to MRI space

We extracted gene expression microarray data from the Allen Institute for Brain Science (AIBS) transcriptome atlas (Hawrylycz *et al.*, 2015). This atlas contains a database of expression levels of 20 737 genes represented by 58 692 probes across the complete cortical mantle and is constructed post-mortem from the brains of six human donors with no history of psychiatric or neuropathological disorders. These data, and details on the methodology of the atlas creation, are freely available to download from the AIBS (http://human.brain-map.org/static/download). Data from all six donors are available for the left hemisphere but only data from two donors are available for the right hemisphere; therefore, only samples of the left hemisphere were included for analysis (180 regions), in accordance with other studies using the AHBA (Romme *et al.*, 2017; McColgan *et al.*, 2018). We used the recently described rigorous method of preprocessing by Arnatkevičiūtė *et al.* (2019) to extract gene expression data from the AHBA and map them to the 180 cortical regions of the Glasser atlas, using code freely available at https://github.com/BMHLab/AHBAprocessing. The methodology of processing steps has been extensively described (Arnatkevičiūtė *et al.*, 2019). In brief, each tissue sample was assigned to an anatomical structure of the 180 left cortical regions of the Glasser atlas, using the AHBA MRI data for each donor. Distances between samples were evaluated on the cortical surface of the left hemisphere, using a 2-mm distance threshold. Probe to gene annotations were updated in Re-Annotator package (Arloth *et al.*, 2015). Only probes where expression measures were above a background threshold in more than 50% of samples were selected. A representative probe for a gene was selected based on highest intensity. Gene expression data were normalized across the left cortex using scaled, outlier-robust sigmoid normalization. Regional expression levels for each gene were compiled to form a 180 × 15745 regional transcription matrix (Arnatkevičiūtė *et al.*, 2019). (Fig. 1C).

### Statistical analysis

#### Demographic, clinical assessments and individual network metrics

Demographics, clinical characteristics and network metrics were compared between the three clinical groups using ANOVA with *post hoc* Tukey for normally distributed and Kruskal-Wallis for non-normally distributed variables. We assessed normality using the Shapiro-Wilk test. For comparisons between PD-VH and PD-non-VH we performed *t*-tests for normally distributed, and Mann-Whitney for non-normally distributed variables. Statistical significance was defined as *P < *0.05. Analyses were performed in Python 3 (Jupyter Lab v1.0.2).

#### Network based statistics

We performed a network-based statistic analysis to investigate whether the presence of visual hallucinations was associated with altered connectivity strength in a subnetwork of the brain (Zalesky *et al.*, 2010). Network-based statistics is a non-parametric connectome-wide analysis used to identify connections and networks comprising the human connectome that are associated with an experimental effect or a between-group difference (Zalesky *et al.*, 2010). A general linear model was used with contrast of interest including PD-VH versus PD-non-VH and Parkinson’s disease versus controls; age and total intracranial volume were included as covariates. Results were replicated for the PD-VH versus PD-non-VH comparison using age, gender and total intracranial volume as covariates as well as correcting for LEDD. Permutation testing with unpaired *t*-tests was performed with 5000 permutations, calculating a test statistic for each connection. A threshold of *t *=* *3.1 as well as family-wise error rate (FWE) of *P < *0.05 was applied (Fig. 1B).

#### Gene co-expression analysis

Co-expression analysis identifies modules of highly co-expressed genes that form a gene co-expression network; co-expressed genes can be thought of as part of the same functional subsystem (Carpenter and Sabatini, 2004; Oldham *et al.*, 2008). Gene co-expression networks are powerful tools in understanding complex genetic interactions in a specific condition, moving from a single gene to a wider molecular pathway or biological process approach; co-expression analyses have already provided significant insights in neurodegeneration (Forabosco *et al.*, 2013; Miller *et al.*, 2013; Bettencourt *et al.*, 2014). Weighted gene co-expression network analysis (WGCNA) is one of the most widely used and validated methods of constructing gene co-expression networks and has been previously described in detail (Langfelder and Horvath, 2008; Langfelder *et al.*, 2011; Botía *et al.*, 2017). In brief, WGCNA uses measures of gene co-expression similarity to construct a network of gene-to-gene co-expression; this can be represented as an nxn matrix for *n* number of genes, where each connection between two genes represents the interaction strength between them. This matrix is then transformed using topological overlap into a proximity matrix where a pair of genes has a high proximity if it is closely interconnected; this way clusters or modules of highly interconnected genes that are co-expressed can be identified.

We performed weighted gene co-expression analysis, using the WGCNA package in R (Zhang and Horvath, 2005; Langfelder and Horvath, 2008) and post-processing with k-means (Botía *et al.*, 2017). Only left hemisphere cortical regions were included in this analysis (180 regions/nodes). We used gene expression data of the left hemisphere from the AHBA for each left cortical brain region of our brain parcellation (180 nodes) (Hawrylycz *et al.*, 2015) with each region or node representing a different sample to construct a gene co-expression network of the healthy brain for our brain parcellation. The nodes/samples that participated in the VH subnetwork were classified as nodes/samples that had the trait of VH whilst the others were classified as non-VH nodes/samples. We assessed for outliers using distance-based networks (Zhang and Horvath, 2005) and, as suggested by the WGCNA authors (Langfelder and Horvath, 2008), we assessed individual genes for expression variance and samples (nodes) for missing entries; three nodes had >50% missing entries and were excluded from further analysis.

Following module identification, we calculated the module membership for each gene within a given module. This is defined as the Pearson's correlation coefficient between gene expression values and the module eigengene and has values between 0 and 1. A value of 1 indicates that a gene’s expression is highly correlated with the module eigengene (or first principal component of a module). Genes with higher module memberships are more representative of the module’s overall function and more likely to be critical components (Fig. 1C).

We then correlated the summary profile (eigengene) for each module to the VH trait using biweight midcorrelation to identify modules significantly associated with the VH subnetwork. For those modules significantly associated following FDR correction (VH-associated modules), we calculated gene significance for the VH trait for each gene of each module. Gene significance is defined as the absolute value of the correlation between the gene and the trait and can be considered as the association of individual genes with clinical information, in our case reduced connectivity in PD-VH. We performed enrichment analysis of the VH-associated modules by ranking the genes of these modules according to their gene significance (Fig. 1D).

#### Gene ontology enrichment analysis

We performed enrichment analysis for gene ontology (GO) and Kyoto Encyclopedia of Genes and Genomes (KEGG) pathway terms for VH-associated modules on g:Profiler (Raudvere *et al.*, 2019) using Benjamini-Hochberg correction for multiple comparisons and significance threshold 0.01. We used the reduce and visualize gene ontology tool (REVIGO) to visualize significant GO terms using semantic similarity (Supek *et al.*, 2011) (Fig. 1D).

#### Expression-weighted cell-type enrichment analysis

We performed expression-weighted cell-type enrichment analysis (EWCE) to determine whether genes within the VH-associated modules have higher expression within a particular cell type than expected by chance (Skene and Grant, 2016). Target lists comprised the genes of VH-associated modules significantly associated with node’s presence in the VH subnetwork (q < 0.05, ranked according to gene significance). Each was run with 100 000 bootstrap lists, controlling for transcript length and content with Benjamini-Hochberg correction for multiple comparisons. Single-cell transcription data were used from the AIBS (https://portal.brain-map.org/atlases-and-data/rnaseq) containing data from the middle temporal gyrus (Hawrylycz *et al.*, 2015). To ensure that our results were not dependent on the dataset used, we replicated our EWCE analysis, with the same parameters (100 000 bootstrap lists, Benjamini-Hochberg correction), using a different human derived dataset from the Regev group (Habib *et al.*, 2017); this is a comprehensive human derived post-mortem datasets, containing data from five donors and 19 550 cells from both the hippocampus and the prefrontal cortex. The EWCE package is freely available here: https://github.com/NathanSkene/EWCE.

### Data availability

Analyses performed in this study used publicly available packages and code (see Supplementary material for details). All data generated from this study are presented in the Supplementary material. Patient-level data will be made available upon request from the authors.

## Results

The study comprised 134 participants: 100 patients with Parkinson’s disease and 34 controls. Of the patients with Parkinson’s disease, 19 were hallucinators (PD-VH) and 81 were not (PD-non-VH). There was no difference in the use of dopamine agonists or amantadine nor in the LEDD between PD-VH and PD-non-VH participants. No participants were receiving antipsychotic medications, acetylcholinesterase inhibitors or anticholinergics at the time of the study. Demographic and clinical details are provided in Table 1. Details on the experienced hallucinatory phenomena are provided in Table 2.

**Table 1:** Demographics and clinical assessments in patients with PD-VH and PD-non-VH patients

Attribute	Controls *n *=* *34	PD-non-VH *n = *81	PD-VH *n = *19	Statistic	*P*-value
Demographics					
Age, years	66.4 (9.3)	64.4 (7.8)	64.6 (8.2)	r^2^ = 0.003	0.459
Male (%)	16 (47.1)	47 (58.0)	6 (31.6)	r^2^ = 0.022	0.086
Years in education	17.6 (2.3)	16.9 (2.7)	17.1 (3.5)	r^2^ = 0.004	0.490
Total intracranial volume, ml	**1397.3 (106.4)**	**1476.4 (130.8)**	**1409.9 (106.7)**	**r^2^ = 0.070**	**0.003** ^*^ ^,^ ^***^
Mood (HADS)					
Depression score	1.6 (2.0)	3.8 (2.9)	4.8 (3.2)	r^2^ = 0.120	<0.001^***^
Anxiety score	**3.8 (3.5)**	**5.6 (3.8)**	**7.7 (4.9)**	r^2^ = **0.071**	**0.0031** ^*^ ^,^ ^**^ ^,^ ^***^
Vision					
LogMAR, best^a^	−0.08 (0.23)	−0.08 (0.16)	−0.06 (0.15)	r^2^ = 0.013	0.854
Pelli Robson, best^a^	1.79 (0.2)	1.79 (0.2)	1.70 (0.2)	r^2^ = 0.016	0.127
D15, total error score	1.29 (1.2)	1.28 (1.1)	1.56 (1.6)	r^2^ = 0.010	0.689
Cognition					
MMSE	29.0 (1.0)	28.9 (1.1)	28.6 (1.8)	r^2^ = 0.004	0.485
MoCA	28.8 (1.3)	28.0 (2.1)	26.9 (3.1)	r^2^ = 0.051	0.011^***^
Disease specific measures					
UPDRS	–	**42.4 (20.2)**	**63.5 (35.6)**	**U = 444**	**0.004**
UPDRS part 3 (motor score)	–	21.8 (11.2)	29.2 (20.8)	U = 604	0.129
UM-PDHQ (hallucination severity score)	–	**0**	**4.4 (2.3)**	–	–
LEDD, mg	–	456.9 (265.0)	434.9 (210.3)	U = 787	0.948
Dopamine agonist use (%)	–	48 (59.3)	9 (47.4)	χ^2^ = 39.59	0.999
Amantadine use (%)	–	8 (9.8)	1 (5.3)	χ^2^ = 57.09	0.998
Disease duration	–	4.0 (2.5)	4.8 (3.4)	U = 669.5	0.339
Sniffin’ sticks	–	7.8 (3.1)	6.1 (3.4)	U = 940.5	0.159
RBDSQ	–	**4.0 (2.5)**	**5.6 (2.5)**	**U = 486**	**0.010**

All data shown are mean (SD) except where stated otherwise. Characteristics that significantly differed between the PD-VH and PD-non-VH are highlighted in bold.

* Significant difference between PD-VH and PD-non-VH.

** Significant difference between PD-non-VH and controls.

*** Significant difference between PD-VH and controls.

^a^ Best binocular score used; LogMAR: lower score implies better performance, Pelli Robson: higher score implies better performance.

HADS = Hospital Anxiety and Depression Scale; LEDD = total levodopa equivalent dose; MMSE = Mini-Mental State Examination; MoCA = Montreal Cognitive Assessment; RBDSQ = REM Sleep Behaviour Disorder Screening Questionnaire; UM-PDHQ = University of Miami Hallucinations Questionnaire (max score = 14); UPDRS = Unified Parkinson’s Disease Rating Scale.

**Table 2:** Characteristics of hallucinations experienced by PD-VH patients

Hallucination characteristics	PD-VH (*n *=* *19)
Phenotype	
Complex hallucinations	11 (57.9%)
Minor hallucinations	8 (42.1%)
Frequency	
<1 a week	11 (57.9%)
>1 a week	8 (42.1%)
Duration	
<1 s	8 (42.1%)
<10 s	6 (31.6%)
>10 s	5 (26.3%)
Insight	
Always preserved	13 (68.4%)
Sometimes preserved	4 (21.1%)
No insight	2 (10.5%)
Number of experienced images mean (SD)	1 (0.67)
Distress	
No distress	14 (73.7%)
Mild to moderate distress	5 (26.3%)

Participants were asked to reflect on all hallucinatory phenomena experienced within the previous month. Complex hallucinations included well form imagery (people, animals, etc), stationary or animate Minor hallucinations included passage hallucinations as well as non-formed images (shadows etc).

### Regional but not global network topology differs in patients with Parkinson’s disease and visual hallucinations

**Figure 2:**
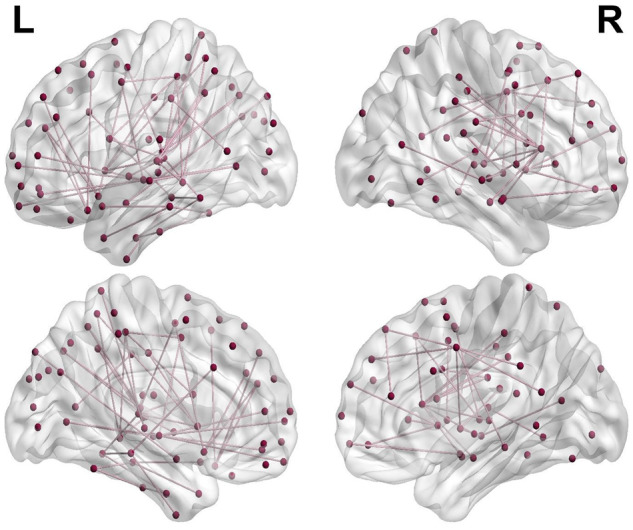
**The VH subnetwork.** Network based statistical analysis revealed a subnetwork of reduced connectivity strength in PD = VH patients, which comprised 92 edges and 82 nodes. The subnetwork was visualized using BrainNetViewer (Xia *et al.*, 2013).

Global network metrics (clustering coefficient and characteristic path length, and density) did not significantly differ between Parkinson’s disease and controls, or PD-VH and PD-non-VH. However, at a regional level, network-based statistics revealed a subnetwork of reduced structural connectivity strength in PD-VH compared to PD-non-VH participants (VH-subnetwork). The subnetwork comprised 92 edges and 82 nodes, controlling for age and total intracranial volume, *P < *0.05. The identified subnetwork with reduced connectivity strength in PD-VH is shown in Fig. 2. A list of all significant connections within the subnetwork is seen in Supplementary Table 1.

No significant subnetwork was identified in the opposite direction (positive correlation between hallucinations and connectivity strength). Importantly our findings cannot be purely attributed to higher disease severity, as no significant subnetwork was identified in Parkinson’s disease compared to controls in either direction nor in Parkinson’s disease participants in relation to total UPDRS, motor UPDRS or MoCA.

### Reduced average controllability is correlated with the presence of hallucinations in Parkinson’s disease

**Figure 3:**
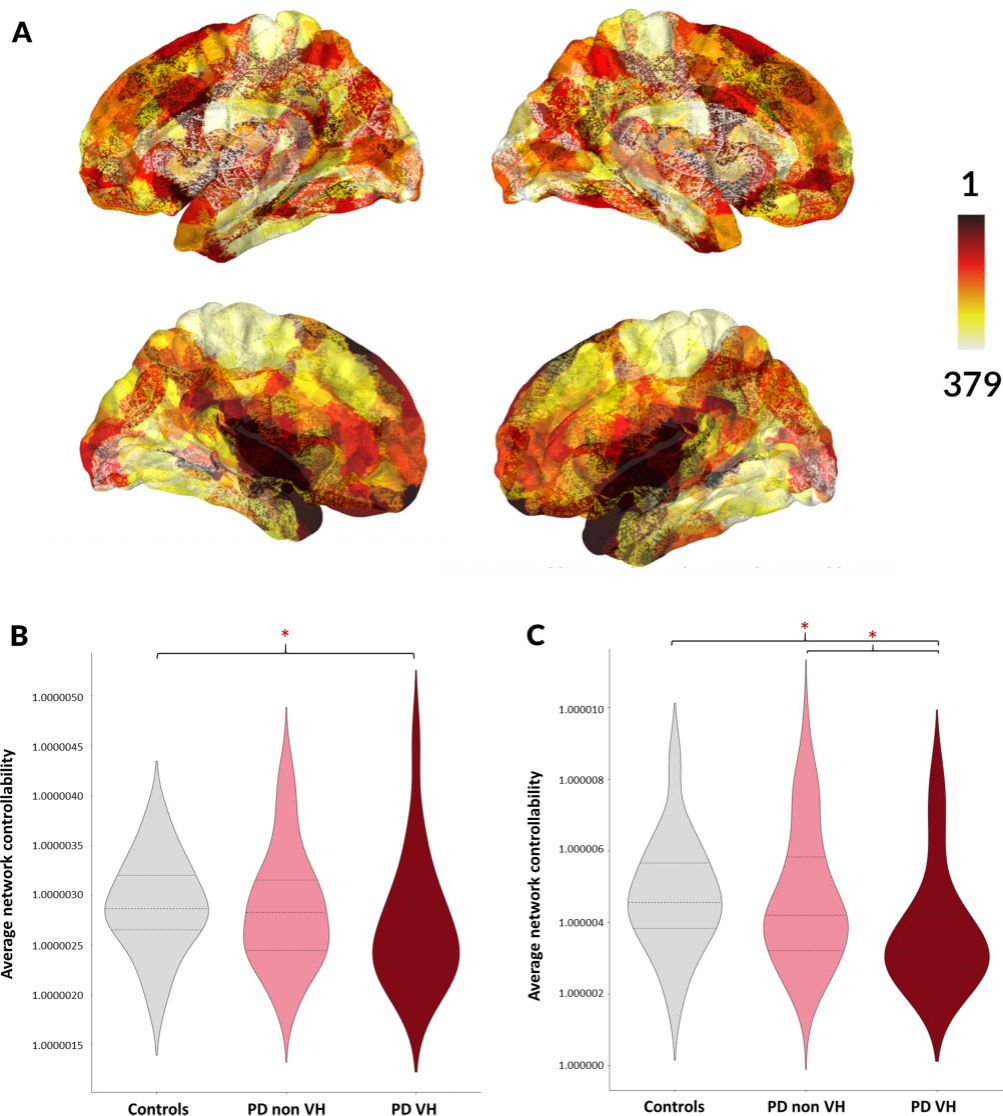
**Reduced controllability in patients with Parkinson’s and hallucinations.** (**A**) Controllability ranking across control participants, visualized using PySurfer (https://pysurfer.github.io/). (**B**) Average controllability in the whole brain network in control participants, PD-non-VH patients PD-VH patients. (**C**) Average controllability in the VH-subnetwork in control participants, PD-non-VH and PD-VH.

First, we assessed average controllability in healthy controls. As previously described (Bernhardt *et al.*, 2019), the thalamus and temporal and prefrontal regions bilaterally were the highest in controllability rank (Fig. 3A; see Supplementary Table 1 for a list of rankings). There was a significant correlation between average controllability and degree strength for each node (U = 568, *P < *0.001), as previously described (Gu *et al.*, 2015; Bernhardt *et al.*, 2019) (Supplementary Fig. 1).

Within the VH-subnetwork, average controllability was significantly reduced in PD-VH compared to both controls (Mann-Whitney: U = 176.5, *P = *0.003) and PD-non-VH (U = 526, *P = *0.014) (Fig. 3C). In contrast, when assessing average controllability across the whole brain network, differences in controllability in PD-VH were less pronounced (PD-VH versus controls U = 233.5, *P = *0.049; PD-VH versus PD-non-VH (U = 625, *P = *0.091) (Fig. 3B). Finally, nodes with higher average controllability in healthy controls were significantly more likely to be within the VH subnetwork (U = 572.5, *P < *0.001) (Supplementary material and Supplementary Fig. 2).

### Gene co-expression patterns linked with presence of hallucinations in Parkinson’s disease

Next, we assessed whether gene co-expression patterns differed in nodes of the VH-subnetwork from the rest of the brain. Gene co-expression networks of the left hemisphere of the healthy adult brain were constructed from the AHBA (Hawrylycz *et al.*, 2015). This resulted in a gene network of 27 modules with gene size ranging from 56 to 1735 (mean = 583, standard deviation = 564). We correlated the identified modules to the presence of the node in the VH-subnetwork (Supplementary Fig. 3). Two modules were significantly correlated (VH-associated modules): the ‘cyan’ module had a negative correlation with the VH-subnetwork (*r* = −0.183, FDR corrected *P*-value: q = 0.014) and the ‘greenyellow’ module had a positive correlation (*r *=* *0.161, q = 0.032). The two modules had gene sizes of 284 and 601, respectively. See the Supplementary material for a complete list of genes included in the two modules.

For both the VH-associated modules, gene module membership was highly correlated with gene significance for the VH-subnetwork (Supplementary Fig. 3); this allowed the ranking of these genes according to gene module memberships for the two modules in subsequent enrichment analyses.

### Functional properties of the visual hallucination-associated modules

**Figure 4:**
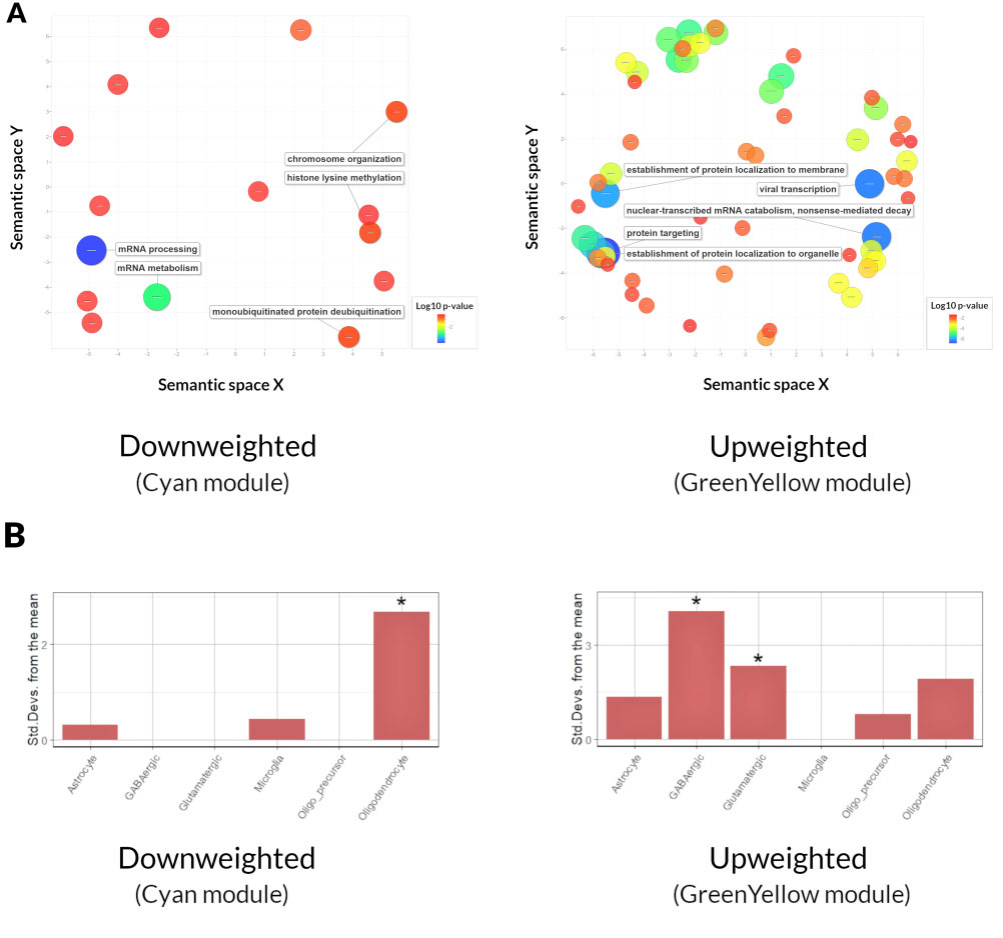
**Gene expression patterns associated with the VH subnetwork.** (**A**) Significant GO terms for biological processes plotted in semantic space, where similar terms are clustered together. The top five most significant GO terms are labelled for each analysis. Redundant GO terms have been excluded. Markers are scaled based on the log10 q-value for the significance of each GO term. Large blue circles are highly significant, while red circles are less significant (see colour bar). (**B**) EWCE for the VH-associated modules using the AIBS dataset. Data are presented as standard deviations from the mean. *Statistically significant (FDR corrected) results.

We performed GO analysis for genes within the VH-associated modules. For the ‘cyan’ module, which had a negative correlation with VH (down-weighted), most significant GO terms included mRNA processing and metabolism, chromosome organization, and histone lysine methylation. In contrast, for the ‘greenyellow’ module that had a positive association with VH (up-weighted), the most significant GO terms included protein localization to membrane and organelle, protein targeting, mRNA catabolism and viral transcription. Enrichment analysis using the KEGG database showed that the ‘greenyellow’ module was significantly enriched in terms related to ribosome (KEGG:03010, q < 0.000, B = 134, *n *=* *522, b = 27); there were no statistically significant KEGG terms for the ‘cyan’ module. The five most significantly enriched GO terms for VH-associated modules are provided in Fig. 4A and Supplementary Table 2), whilst the full list of significant GO terms are provided in Supplementary Table 4.

We then investigated whether the genes (ranked by gene significance) within the VH-associated modules were enriched in specific cell types. For the down-weighted ‘cyan’ module, we saw an enrichment in oligodendrocytes (Fig. 4B). In contrast, up-weighted genes within the ‘greenyellow’ module were enriched in glutamatergic neurons and GABAergic interneurons (Fig. 4B). To ensure that cell type enrichment results were not influenced by the dataset used, we replicated our results using data from the Regev group (Habib *et al.*, 2017). We saw a similar enrichment pattern: top genes of the down-weighted ‘cyan’ module were enriched for oligodendrocyte markers and genes of the upweighted ‘greenyellow’ module were enriched for neuronal cells and GABAergic interneurons (Supplementary Fig. 4).

## Discussion

We used controllability analysis to show that Parkinson’s disease hallucinations are associated with structural connectivity changes in brain regions that exert the greatest control over the whole brain network, and linked these changes with different underlying patterns of regional gene expression. Specifically: (i) we identify a subnetwork of reduced structural connectivity in PD-VH; (ii) this subnetwork is critical for brain integration and hallucinations, as nodes with high controllability in controls are more likely to participate in this subnetwork and controllability is reduced within this subnetwork in patients with hallucinations; (iii) we show that regional gene expression in areas within the affected subnetwork have a characteristic pattern with down-weighted genes related to mRNA metabolism, chromosome organization and histone lysine methylation and up-weighted genes related to protein targeting and localization; and (iv) down-weighted genes are enriched in oligodendrocyte markers, and up-weighted genes in glutamatergic neurons and GABAergic interneurons.

Our finding that regions with high controllability are particularly affected in Parkinson’s disease hallucinations is consistent with current models that implicate shifts in integration of different brain networks, specifically dysfunction in attentional brain networks (Muller *et al.*, 2014), with overactivity of the DMN and impaired dorsal attention network involvement (Shine *et al.*, 2014, 2015; Yao *et al.*, 2014; Baggio *et al.*, 2015). By showing loss of a structural network with normally high controllability, we provide structural evidence to support this model.

Loss of structural connectivity was recently shown in patients with Parkinson’s disease susceptible to visual illusions, preferentially involving highly connecting nodes (Hall *et al.*, 2019). Our findings extend that work by showing that the affected subnetwork exerts high levels of control across the brain, and a selective vulnerability underlies white matter connectivity loss in Parkinson’s disease.

Other recent models for hallucinations in Parkinson’s disease strongly implicate thalamic regions, potentially as drivers of these shifts in network control (Onofrj *et al.*, 2017, 2019), with converging evidence for thalamic involvement, especially lateral geniculate networks, identified using recent network localization techniques (Kim *et al.*, 2019; Weil *et al.*, 2019). Indeed, in the analysis presented here, both the right and left thalamus participate in the VH-subnetwork, and is amongst the areas with the highest controllability ranking, suggesting that this region has significant influence over whole brain function.

Our observation that white matter connectivity loss in PD-VH is linked to specific regional gene expression patterns provides mechanistic insights into the observed structural connectivity changes in Parkinson’s disease hallucinations. We found a pattern of downweighted histone lysine methylation and genes related to mRNA processing associated with the subnetwork affected in Parkinson’s disease hallucinations. Histone methylation is important for transcriptional control (Greer and Shi, 2012) and closely related to DNA methylation (Cedar and Bergman, 2009), which is a hallmark of ageing, predicting lifespan (Hannum *et al.*, 2013; Marioni *et al.*, 2015; Michalak *et al.*, 2019). RNA segments have also been shown to accumulate in ageing neurons (Sudmant *et al.*, 2018; Butler *et al.*, 2019) whilst, recently, impaired nucleic acids repair has been implicated as an age-related modifier of Parkinson’s disease (Sepe *et al.*, 2016). Brain regions with reduced expression of genes related to histone methylation and RNA processing could be more vulnerable to such ageing-related changes.

We also found that genes of the up-weighted greenyellow module were enriched in genes related to protein localization, both intracellularly and at the cell membrane in regions of connectivity loss in PD-VH. An important mechanism for degeneration in Parkinson’s disease is thought to be dysfunction in the autophagy-lysosome pathway (ALP) (Pan *et al.*, 2008), particularly within its subpathway, chaperone-mediated autophagy (Alvarez-Erviti *et al.*, 2011), with lysosomal malfunction leading to accumulation of alpha-synuclein (Cuervo *et al.*, 2010; Lawrence and Zoncu, 2019). Mutations in *GBA* are associated with a higher risk of Parkinson’s disease whilst even in sporadic Parkinson’s disease glucocerebrosidase activity is significantly decreased, with associated impaired lysosomal chaperone-mediated authophagy (Murphy *et al.*, 2014). Patients with *GBA*-related Parkinson’s disease also have a higher rate of hallucinations (Neumann *et al.*, 2009; Brockmann *et al.*, 2011), whilst the most common *GBA*-mutations associated with Parkinson’s disease (N370S and L44P; Velayati *et al.*, 2010) are thought to induce endoplasmic reticulum stress through activation of the unfolded protein response (Mu *et al.*, 2008; Doyle *et al.*, 2011; Sanchez-Martinez *et al.*, 2016). Our finding of higher enrichment in membrane and organelle localization genes as well as the presence macro-autophagy amongst significantly enriched GO terms of the up-weighted module (Supplementary Table 4) provide further support to the key role of the ALP in Parkinson’s disease, particularly in the presence of hallucinations.

Regional changes in gene expression can be explained by different cell populations (preferentially expressing different genes) being expressed in different numbers across brain regions. Thus, we assessed whether genes associated with connectivity loss in PD-VH were preferentially enriched in different cell types. We found that loss of structural connectivity in PD-VH was associated with down-weighted genes enriched in oligodendrocytes and up-weighted genes enriched in neuronal cells. Oligodendrocytes have recently been implicated in Parkinson’s disease, with heritability for Parkinson’s disease enriched in oligodendrocyte-specific genes (Bryois *et al*., 2020). The observed changes in structural connectivity in PD-VH included multiple long connections between spatially remote areas. Longer connections are likely to rely more on myelination for signal transfer than shorter connections, as oligodendrocytes play a key role in myelination, myelin remodelling, regulation of conduction velocity and axonal metabolic support (Young *et al.*, 2013; Pepper *et al.*, 2018). Regions where oligodendrocytes are less expressed, may therefore be more vulnerable to connectivity loss.

Several methodological considerations should be taken into account when interpreting the results of our study. Our findings are built on structural data determined with diffusion tractography. Limitations of this method include uncertainty for crossing fibres. We used multi-shell data, and improving post-processing techniques [including constrained spherical deconvolution (Tournier *et al.*, 2007) and the SIFT2 algorithm (Smith *et al.*, 2015)], in order to provide the best possible estimate of underlying structural connectivity. Using gene expression data from healthy human brains to understand transcription changes in Parkinson’s disease could be limited if transcription in Parkinson’s disease was different from healthy brains. Our main comparison of interest was between PD-VH and PD-non-VH; so that even if cortical gene expression differed significantly in Parkinson’s disease compared to healthy brains, we would expect these Parkinson’s disease-related changes to be similar in both groups. In addition, a recent study has confirmed higher expression of known genetic risk factors for Parkinson’s disease in regions involved in Braak Lewy body stages in the Allen donors, suggesting that data from non-neurological adults can provide useful insights into selective vulnerability in Parkinson’s disease (Keo *et al.*, 2020). Nevertheless, it is still possible that differences in cortical gene expression between Parkinson’s disease and controls have a significant influence and clarifying potential transcriptome changes in Parkinson’s disease using brain tissue of patients with and without hallucinations could be an area of future research. Although comparable to other studies of Parkinson’s disease hallucinations (Yao *et al.*, 2014; Hepp *et al.*, 2017; Hall *et al.*, 2019) the sample size for PD-VH participants remains small and our three study groups differ in size; replication of our results in larger cohorts as well as longitudinal assessment of connectivity changes in Parkinson’s disease hallucinations would provide further insights. All participants with Parkinson’s disease were scanned on their usual dopaminergic medications. Although we think it is unlikely that dopaminergic medication would affect structural connectivity, as corrected fractional anisotropy is not affected by levodopa (Chung *et al.*, 2017), further research could clarify possible effect of dopaminergic medications in diffusion-derived metrics.

## Conclusions

We show that visual hallucinations in Parkinson’s disease are associated with the breakdown of a structural subnetwork that possesses distinct gene expression patterns and cellular subtypes and exerts control across distributed brain regions. Our findings provide insights into how hallucinations develop in Parkinson’s disease and indicate potential targets for future therapeutic trials.

## Funding

A.Z. is supported by an Alzheimer’s Research UK Clinical Research Fellowship (2018B-001). P.M.C. is supported by the National Institute for Health Research. R.S.W. is supported by a Wellcome Clinical Research Career Development Fellowship (201567/Z/16/Z). We gratefully acknowledge the support of NVIDIA Corporation with the donation of the Quadro P6000 GPU used for this research.

## Competing interests

The authors report no competing interests.

## Supplementary material

Supplementary material is available at *Brain* online.

## Supplementary Material

awaa270_Supplementary_Data

## Abbreviations

AHBA =: Allen Human Brain Atlas
EWCE =: expression weighted cell enrichment
GO =: gene ontology
PD-non-VH =: Parkinson's disease patients without hallucinations
PD-VH =: Parkinson’s disease patients with visual hallucinations
VH =: visual hallucinations
