# Supplementary material for: Differences in network controllability and regional gene expression underlie hallucinations in Parkinson’s disease
Source: Brain. 2020 Oct 29;143(11):3435–48. doi: 10.1093/brain/awaa270 (PMC7719028; doi:10.1093/brain/awaa270)
Supplement: awaa270_Supplementary_Data [file awaa270_supplementary_data.zip › awaa270-suppl_data/brain-2019-02293-File006.pdf]

Supplementary Material

Differences in network controllability and regional gene expression underlie visual hallucinations in Parkinson's disease

Angeliki Zarkali, Peter McColgan, Mina Ryten, Regina Reynolds, Louise-Ann Leyland, Andrew J. Lees, Geraint Rees, Rimona S. Weil

Contents

Supplementary Results 2

Table S1. Significant connections in the subnetwork of reduced connectivity in patients with Parkinson's and visual hallucinations (VH-subnetwork). 2

Table S2. Cortical and subcortical regions and their rank controllability in controls 3

Table S3. Gene Ontology (GO) terms for biological processes associated with VH-associated modules. 5

Figure S1. Structural connectome density in patients with Parkinson's disease with and without hallucinations and controls. 6

Figure S2. Relationship between controllability ranking and degree strength. 7

Figure S4. Subnetwork of reduced connectivity strength in PD-VH using age, gender, and total intracranial volume as covariates. 9

Figure S5. Co-expression network analysis and correlation with the VH-subnetwork. 10

Figure S6: Cell type enrichment of topgenes for downweighted and upweighted VH-associated modules. 11

Additional Files 12

URLs 12

References 12

Supplementary Results

Table S1. Significant connections in the subnetwork of reduced connectivity in patients with Parkinson's and visual hallucinations (VH-subnetwork).

L_ventral_23ab to L_p24pr. Test stat: 3.73<br>L_p24pr to L_8BL. Test stat: 3.15<br>L_p24pr to L_9p. Test stat: 3.32<br>L_2 to L_44. Test stat: 3.57<br>L_8Av to L_44. Test stat: 3.12<br>L_44 to L_52. Test stat: 3.12<br>L_a47r to L_MiddleInsular. Test stat: 3.12<br>L_55b to L_FrontalOPercular_1. Test stat: 3.19<br>L_44 to L_PFt. Test stat: 3.44<br>L_p24pr to L_PreSubiculum. Test stat: 3.21<br>L_PoI2 to L_AuditoryComplex_5. Test stat: 3.13<br>L_AuditoryComplex_5 to L_TemporoParietoOccipitalJunction_3. Test stat: 3.22<br>L_6_ventral to L_PF_opercular. Test stat: 3.17<br>L_44 to L_PF_opercular. Test stat: 3.13<br>L_44 to L_PF. Test stat: 3.25<br>L_6r to L_PF. Test stat: 3.22<br>L_44 to L_PosteriorInsular_1. Test stat: 3.11<br>L_MiddleInsular to L_FrontalOpercular_5. Test stat: 3.26<br>L_44 to L_ParaInsular. Test stat: 3.27<br>L_a47r to L_ParaInsular. Test stat: 3.16<br>L_8BL to R_p24. Test stat: 3.26<br>L_V8 to R_V6. Test stat: 3.10<br>L_ProStriate to R_V6. Test stat: 3.45<br>L_ProStriate to R_V3A. Test stat: 3.25<br>L_5L to R_PrimaryAuditory_1. Test stat: 3.30<br>L_8BL to R_5m_ventral. Test stat: 3.65<br>L_9p to R_SupplementaryCingulateEyeField. Test stat: 3.40<br>L_SupplementaryCingulateEyeField to R_6ma. Test stat: 3.54<br>L_6ma to R_6ma. Test stat: 3.43<br>L_33pr to R_6ma. Test stat: 3.32<br>L_6a to R_6ma. Test stat: 3.46<br>R_PrimaryAuditory_1 to R_LateralIntraParietalVentral. Test stat: 3.12<br>L_SuperiorFrontalLanguage to R_8BM. Test stat: 3.17<br>R_p24 to R_10r. Test stat: 3.22<br>L_PoI2 to R_8BL. Test stat: 3.12<br>R_p24 to R_10d. Test stat: 3.35<br>L_10d to R_a47r. Test stat: 3.27<br>R_p24 to R_a47r. Test stat: 3.14<br>L_VentralIntraParietalComplex to R_6r. Test stat: 3.45<br>R_2 to R_6r. Test stat: 3.17<br>R_p24 to R_9-46d. Test stat: 3.39<br>L_33pr to R_9a. Test stat: 3.11<br>L_10d to R_a10p. Test stat: 3.28<br>R_8Av to R_47s. Test stat: 3.53<br>R_9-46d to R_47s. Test stat: 3.28<br>R_PrimaryAuditory_1 to R_6a. Test stat: 3.17<br>L_SuperiorFrontalLanguage to R_s6-8. Test stat: 3.14	L_6ma to R_s6-8. Test stat: 3.53<br>L_2 to R_s6-8. Test stat: 3.84<br>L_p24pr to R_OP2-3. Test stat: 3.15<br>R_7PC to R_MiddleInsular. Test stat: 3.69<br>R_3a to R_MiddleInsular. Test stat: 3.21<br>R_6a to R_AnteriorAngranularInsulaComplex. Test stat: 3.12<br>R_MedialSuperiorTemporal to R_AuditoryComplex_5. Test stat: 3.22<br>L_VentralIntraParietalComplex to R_STSv_posterior. Test stat: 3.28<br>R_7PC to R_STSv_posterior. Test stat: 3.27<br>L_V3A to R_DorsalTransitionalVisualArea. Test stat: 3.24<br>L_ProStriate to R_DorsalTransitionalVisualArea. Test stat: 3.35<br>R_PrimaryAuditory_1 to R_IntraParietaR_1. Test stat: 3.36<br>L_V8 to R_V6A. Test stat: 3.11<br>L_ProStriate to R_V3CD. Test stat: 3.10<br>R_MedialSuperiorTemporal to R_PosteriorInsular_1. Test stat: 3.10<br>R_55b to R_PosteriorInsular_1. Test stat: 3.14<br>R_a47r to R_PosteriorInsular_1. Test stat: 3.58<br>R_10pp to R_PosteriorInsular_1. Test stat: 3.10<br>R_PoI2 to R_LateralBeltComplex. Test stat: 3.23<br>R_STSv_posterior to R_AuditoryComplex_4. Test stat: 3.13<br>R_IntraParietaR_1 to R_AuditoryComplex_4. Test stat: 3.35<br>R_LateralBeltComplex to R_AuditoryComplex_4. Test stat: 3.16<br>R_V6A to R_ParaInsular. Test stat: 3.12<br>L_55b to L_Cerebellum. Test stat: 3.27<br>L_PresylvianLanguage to L_Cerebellum. Test stat: 3.48<br>L_44 to L_Cerebellum. Test stat: 3.56<br>L_AuditoryComplex_5 to L_Cerebellum. Test stat: 3.24<br>L_PHT to L_Cerebellum. Test stat: 3.52<br>L_AuditoryComplex_4 to L_Cerebellum. Test stat: 3.35<br>R_6r to L_Cerebellum. Test stat: 3.14<br>R_ParaInsular to L_Cerebellum. Test stat: 3.12<br>L_1 to L_Thalamus. Test stat: 3.20<br>L_Cerebellum to L_Thalamus. Test stat: 3.63<br>L_PresylvianLanguage to L_Putamen. Test stat: 3.12<br>L_V8 to Brain-Stem. Test stat: 3.19<br>L_44 to Brain-Stem. Test stat: 3.70<br>L_44 to L_Hippocampus. Test stat: 3.19<br>R_AuditoryComplex_5 to R_Cerebellum. Test stat: 3.25<br>R_5L to R_Thalamus. Test stat: 3.24<br>R_s6-8 to R_Thalamus. Test stat: 3.25<br>R_Cerebellum to R_Thalamus. Test stat: 3.45<br>R_ParaBeltComplex to R_Pallidum. Test stat: 3.19<br>R_STSv_posterior to R_Pallidum. Test stat: 3.52<br>R_AuditoryComplex_4 to R_Pallidum. Test stat: 3.12

Table S2. Cortical and subcortical regions and their rank controllability in controls

1	R_Cerebellum	71	L_PH	141	L_STSd_posterior
2	L_Cerebellum	72	L_PGs	142	L_45
3	L_V2	73	L_9-46d	143	R_6_ventral
4	L_V1	74	L_AuditoryComplex_4	144	R_TE1_middle
5	R_V2	75	R_AuditoryComplex_5	145	L_55b
6	R_V1	76	R_PerirhinalEctorhinalCortex	146	R_TE1_anterior
7	L_V3	77	L_VentralVisualComplex	147	L_SupplemCingulateEyeField
8	R_V3	78	L_posteriorOFC	148	L_TE1_anterior
9	R_Thalamus	79	R_PosteriorInferoTemporal	149	L_VentroMedialVisual_1
10	L_Caudate	80	R_9-46d	150	R_9p
11	L_Thalamus	81	R_PH	151	R_47s
12	L_Putamen	82	L_PerirhinalEctorhinalCortex	152	R_PGp
13	R_Putamen	83	L_AuditoryComplex_5	153	L_7PC
14	R_Caudate	84	R_OrbitoFrontalCortex	154	L_MedialIntralParietal
15	Brain-Stem	85	R_PirformCortex	155	L_9p
16	L_V4	86	L_46	156	L_IFSa
17	R_V4	87	R_FrontalEyeFields	157	L_10v
18	L_PrimaryMotorCortex_4	88	L_Amygdala	158	L_i6-8
19	R_Pallidum	89	L_AnteriorIntraParietal	159	L_OP2-3
20	L_1	90	L_PHT	160	L_IntraParietal_1
21	R_PrimaryMotorCortex_4	91	R_V8	161	R_d32
22	L_Pallidum	92	R_TemporoParietoOccipitalJunction_1	162	R_IntraParietaR_2
23	L_PrimarySenroryCortex_3b	93	R_AuditoryComplex_4	163	L_V6
24	R_1	94	R_5m	164	L_LateralOccipital_2
25	L_2	95	R_8BM	165	L_PFt
26	R_2	96	R_9m	166	L_ProStriate
27	R_VentralDC	97	R_6r	167	L_FrontalOpercular_5
28	L_VentralDC	98	L_8Ad	168	R_SuperiorTemporalVisual
29	R_PrimarySenroryCortex_3b	99	L_47s	169	R_5m_ventral
30	L_3a	100	L_PF	170	R_44
31	L_TF	101	R_8Ad	171	R_VentroMedialVisuaR_3
32	L_6_dorsal	102	R_PGs	172	R_FrontalOpercular_5
33	R_6_dorsal	103	R_ParietoOccipitalSulcus_2	173	L_8BL
34	R_TG_dorsal	104	R_ParetoOccipitalSulcus_1	174	L_47l
35	L_TE2_posterior	105	R_8BL	175	L_PFcm
36	R_TE2_anterior	106	R_6ma	176	L_PreSubiculum
37	R_3a	107	L_11l	177	L_TemporoParietoOccipitalJun_2
38	L_TE2_anterior	108	L_ParaHippocampalArea_3	178	L_FST
39	R_TF	109	R_VentralVisualComplex	179	L_V3CD
40	L_6mp	110	R_p9-46v	180	R_V6
41	R_6mp	111	R_11l	181	R_LateralOccipitaR_2
42	L_TG_dorsal	112	R_OP4	182	R_24d_ventral
43	L_V3A	113	R_AnteriorIntraParietal	183	R_7PC
44	R_V3A	114	R_posteriorOFC	184	R_45
45	R_TE2_posterior	115	L_RertroSplenialCortex	185	R_43
46	L_PFm	116	L_ParetoOccipitalSulcus_1	186	R_PFt
47	L_PGi	117	L_TemporoParietoOccipitalJunction_1	187	R_ProStriate
48	L_TG_ventral	118	L_TE1_middle	188	R_ParaHippocampalArea_3
49	L_TE1_posterior	119	R_PHT	189	L_MedialSuperiorTemporal
50	R_Hippocampus	120	L_Accumbens	190	L_7m
51	L_6a	121	L_ParietoOccipitalSulcus_2	191	L_5m_ventral
52	L_Hippocampus	122	R_TemporoParietoOccipitalJunction_2	192	L_8BM
53	R_6a	123	L_PosteriorInferoTemporal	193	L_10d
54	L_OrbitoFrontalCortex	124	R_SupplementaryCingulateEyeField	194	L_OP4
55	R_8C	125	L_V8	195	L_ParaHippocampalArea_1
56	R_TG_ventral	126	R_VentroMedialVisuaR_1	196	L_STSv_posterior
57	R_Amygdala	127	R_Accumbens	197	L_IntraParietal_2
58	L_13l	128	R_RertroSplenialCortex	198	L_VentroMedialVisual_3
59	R_TE1_posterior	129	L_5m	199	R_SuperiorFrontalLanguage
60	L_8Av	130	R_PF	200	R_p32pr
61	R_FusiformFaceComplex	131	L_9m	201	R_IFSa
62	R_24d_dorsal	132	R_a47r	202	R_9a
63	R_8Av	133	L_a47r	203	R_i6-8
64	L_FrontalEyeFields	134	L_OP1	204	R_OP1
65	R_PGi	135	L_PirformCortex	205	R_STSd_posterior
66	L_24d_dorsal	136	R_10v	206	R_DorsalTransitionalVisualArea
67	L_8C	137	R_13l	207	L_5L
68	R_46	138	L_6ma	208	L_7_lateral_area
69	R_PFm	139	L_p9-46v	209	L_LateralIntraParietalVentral
70	L_FusiformFaceComplex	140	L_9a	210	L_6_ventral

211	L_a9-46v	268	L_PGp	325	R_AnteriorVentralInsularArea
212	L_FrontalOPercular_4	269	L_PF_opercular	326	R_AnteriorAngranInsulaComplex
213	L_EntorhinalCortex	270	L_V6A	327	R_EntorhinalCortex
214	L_TemporoParietoOccipitalJunc_3	271	L_31pd	328	R_PreSubiculum
215	L_p47r	272	L_InsularGranularComplex	329	R_ParaHippocampalArea_1
216	R_MedialIntralParietal	273	L_MedialBeltComplex	330	R_V4t
217	R_10r	274	L_LateralBeltComplex	331	R_31a
218	R_10d	275	R_MedialSuperiorTemporal	332	R_InsularGranularComplex
219	R_10pp	276	R_IntraParietalSulcus_1	333	R_MedialBeltComplex
220	R_FrontalOPercular_4	277	R_LateralOccipitaR_1	334	R_STSv_anterior
221	R_FrontalOPercular_2	278	R_PresylvianLanguage	335	L_7Pm
222	R_STSd_anterior	279	R_ventraR_23ab	336	L_23d
223	R_TemporoParietoOccipitalJun_3	280	R_23c	337	L_dorsal_23ab
224	R_p47r	281	R_5L	338	L_p24pr
225	L_d32	282	R_7_lateraR_area	339	L_33pr
226	L_44	283	R_7A_medial	340	L_a24pr
227	L_ParaBeltComplex	284	R_LateralIntraParietalVentral	341	L_a24
228	L_STSd_anterior	285	R_VentralIntraParietalComplex	342	L_p32
229	L_DorsalTransitionalVisualArea	286	R_IFSp	343	L_IFJp
230	L_p10p	287	R_a10p	344	L_IFSp
231	L_STSv_anterior	288	R_TA2	345	L_52
232	R_OP2-3	289	R_FrontalOPercular_3	346	L_TA2
233	R_PFcm	290	R_ParaBeltComplex	347	L_FrontalOPercular_1
234	R_IntraParietaR_1	291	R_V6A	348	L_FrontalOPercular_3
235	R_PF_opercular	292	R_V3CD	349	L_Hippocampus
236	R_FST	293	R_VentroMedialVisuaR_2	350	L_ParaHippocampalArea_2
237	R_LateralOccipitaR_3	294	R_p10p	351	L_V4t
238	R_25	295	L_PremotorEyeFields	352	L_VentroMedialVisual_2
239	R_a32pr	296	L_IntraParietalSulcus_1	353	L_31a
240	L_V7	297	L_MiddleTemporal	354	L_s32
241	L_V3B	298	L_ventral_23ab	355	L_PosteriorInsular_1
242	L_LateralOccipital_1	299	L_31p_ventral	356	L_ParaInsular
243	L_PrimaryAuditory_1	300	L_23c	357	R_p24
244	L_PresylvianLanguage	301	L_24d_ventral	358	R_PremotorEyeFields
245	L_SuperiorFrontalLanguage	302	L_7A_medial	359	R_PrimaryAuditory_1
246	L_SuperiorTemporalVisual	303	L_10r	360	R_23d
247	L_p32pr	304	L_IFJa	361	R_dorsaR_23ab
248	L_6r	305	L_10pp	362	R_31p_ventral
249	L_a10p	306	L_s6-8	363	R_p24pr
250	L_PoI2	307	L_43	364	R_a24pr
251	L_AnteriorVentralInsularArea	308	L_RetroInsularCortex	365	R_a24
252	L_IntraParietal_0	309	L_MiddleInsular	366	R_47l
253	L_25	310	L_FrontalOPercular_2	367	R_IFJp
254	L_a32pr	311	L_STGa	368	R_LateralIntralParietalDorsal
255	R_V7	312	L_LateralOccipital_3	369	R_52
256	R_7m	313	R_55b	370	R_Hippocampus
257	R_p32	314	R_V3B	371	R_STGa
258	R_47m	315	R_MiddleTemporal	372	R_STSv_posterior
259	R_s6-8	316	R_PrecuneusVisual	373	R_ParaHippocampalArea_2
260	R_FrontalOPercular_1	317	R_7Pm	374	R_31pd
261	R_IntraParietaR_0	318	R_7P_lateral	375	R_s32
262	L_PrecuneusVisual	319	R_33pr	376	R_PosteriorInsular_1
263	L_7P_lateral	320	R_IFJa	377	R_LateralBeltComplex
264	L_VentralIntraParietalComplex	321	R_a9-46v	378	R_ParaInsular
265	L_47m	322	R_RetroInsularCortex	379	R_p24
266	L_LateralIntralParietalDorsal	323	R_PoI2		
267	L_AnteriorAngranInsulaComplex	324	R_MiddleInsular		

Table S3. Gene Ontology (GO) terms for biological processes associated with VH-associated modules.

GO term	Description	q value	B	N	b
**Downweighted Genes (Cyan Module)**					
**GO term**	**Description**	**q value**	**B**	**N**	**b**
GO:0006397	mRNA processing	0.001	517	272	25
GO:0016071	mRNA metabolic process	0.005	860	272	32
GO:0034968	histone lysine methylation	0.035	119	221	8
GO:0035520	monoubiquitinated protein deubiquitination	0.035	10	161	3
GO:0051276	chromosome organization	0.035	1238	180	28
**Upweighted Genes (Greenyellow Module)**					
**GO term**	**Description**	**q value**	**B**	**N**	**b**
GO:0006605	protein targeting	1.38E-07	428	523	41
GO:0019083	viral transcription	5.06E-07	178	519	24
GO:0000184	nuclear-transcribed mRNA catabolic process, nonsense-mediated decay	5.06E-07	122	519	20
GO:0072594	establishment of protein localization to organelle	6.61E-07	565	534	46
GO:0090150	establishment of protein localization to membrane	1.07E-06	330	535	33

The top five most significant GO terms are displayed for each connection type. Redundant GO terms have been removed.
q value: log10 of the FDR adjusted p value; B: Total number of genes associated with a specific GO term;
b:Number of genes in the intersection ; N: Number of genes in the target set (query size).

Figure S1. Structural connectome density in patients with Parkinson's disease with and without hallucinations and controls.

Connectome density ± SD was 0.057 ± 0.092 in PD-VH, 0.611 ± 0.084 in PD-non-VH and 0.588 ± 0.095 in Control participants. Density did not significantly differ between the three groups (r^2=0.015, df=135, p=0.139).

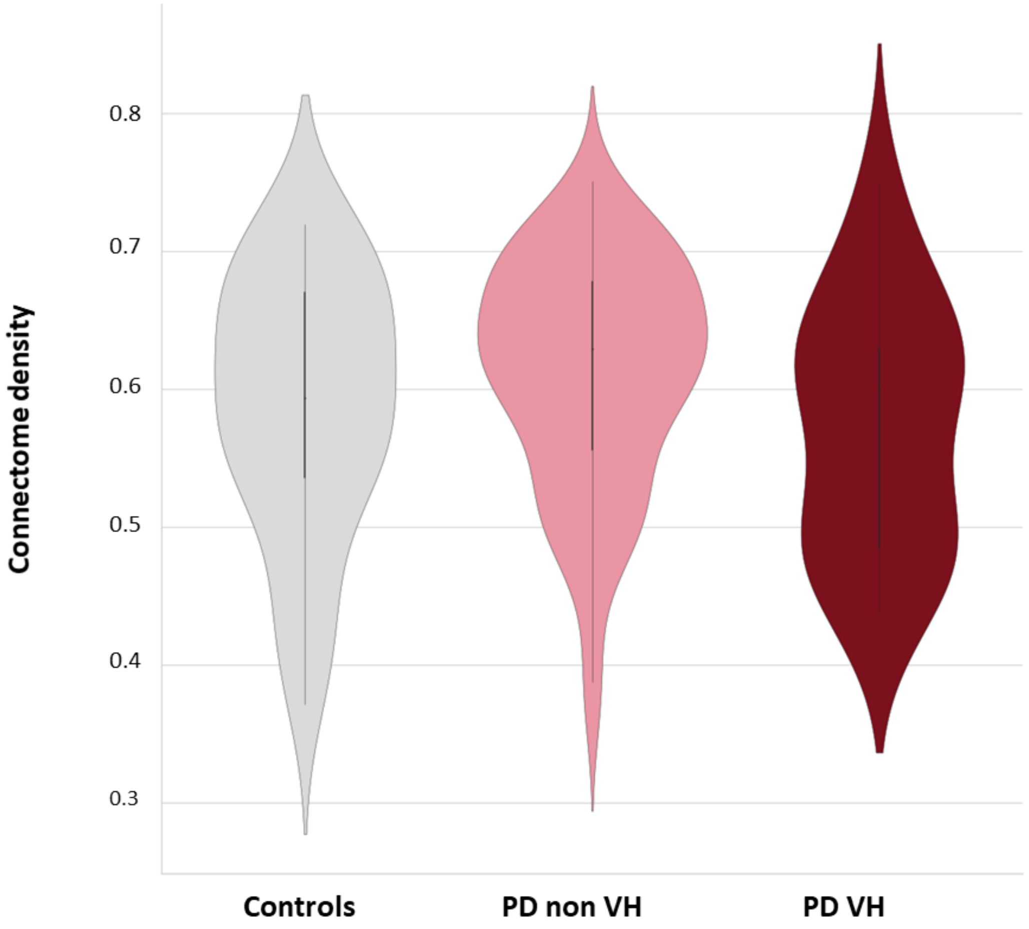

Figure S2. Relationship between controllability ranking and degree strength.

A node's rank in terms of controllability is significantly associated with it's rank according to degree strength, as previously described (Gu *et al.*, 2015, 2017; Bernhardt *et al.*, 2019)

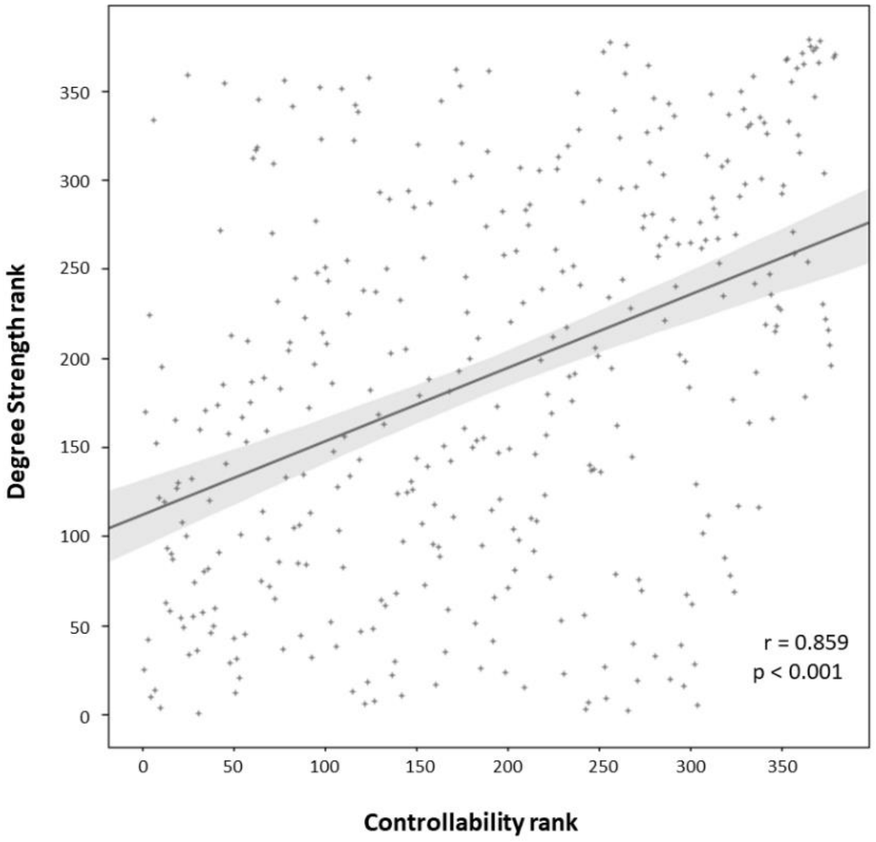

Figure S3. Average controllability of nodes that are participating in the VH-subnetwork and those that are not. Nodes with higher controllability in control participants are more likely to be within the subnetwork (U=572.5, $p<0.001$).

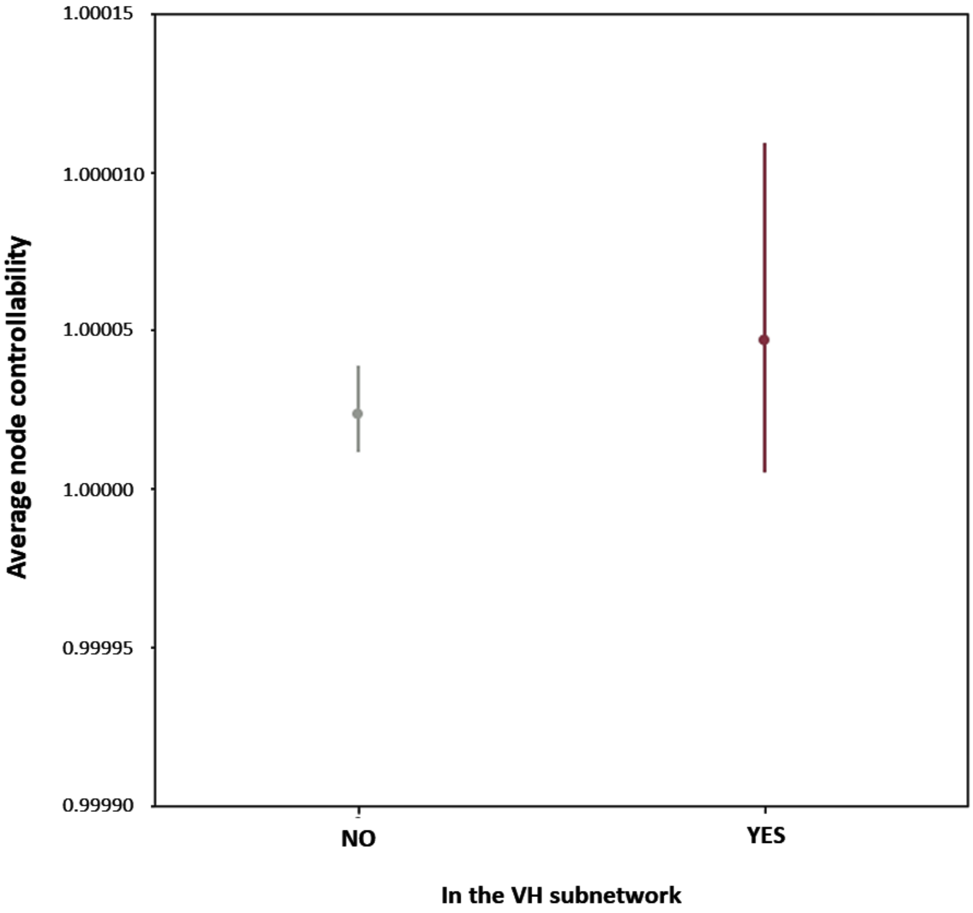

Figure S4. Subnetwork of reduced connectivity strength in PD-VH using age, gender, and total intracranial volume as covariates.

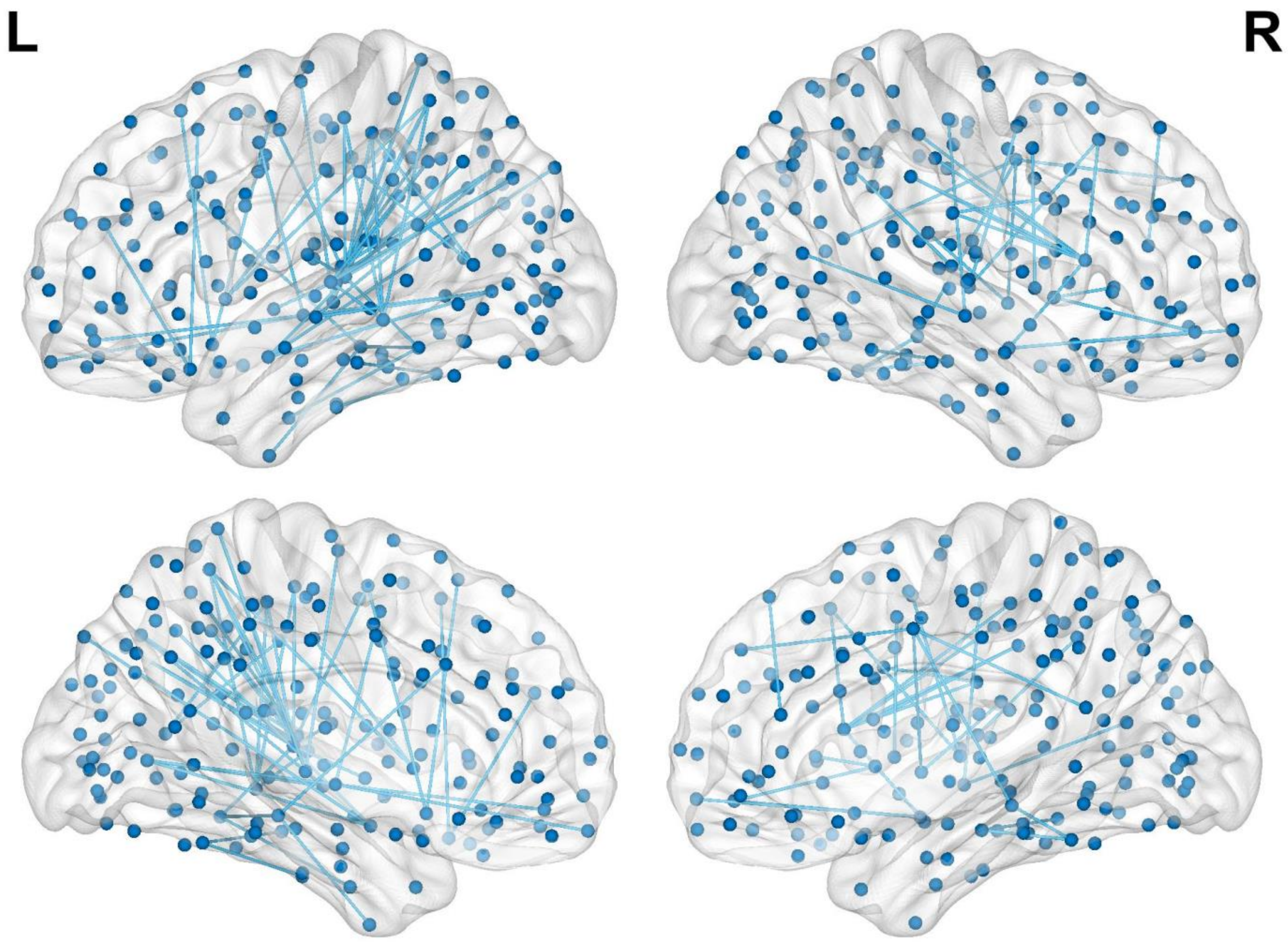

Although PD-VH and PD-non-VH participants did not significantly differ in gender, we repeated network-based statistics with gender included as covariate to ensure stability of our results.

A similar subnetwork of reduced connectivity strength was identified in PD-VH when we included gender; this comprised of 91 nodes and 165 edges. We used the Jaccard similarity index to assess the similarity of the two identified subnetworks (with and without gender as covariate). This is defined as the size of the intersection of two samples A and B, divided by the size of the union of the two sets:

$$J(A,B) = \frac{|A \cap B|}{|A \cup B|}$$

The two subnetworks had a high Jaccard similarity index (0.762). They were particularly similar in terms of participating nodes, and for the left cortical regions (where nodes were included in subsequent genetic analyses) they differed only in 6 nodes which were present in the subnetwork with gender included but not without. These were: L_V1, L_OrbitoFrontalCortex, L_i6-8, L_PreSubiculum, L_Hippocampus, L_ParaHippocampalArea_3 and L_TE2_posterior.

Figure S5. Co-expression network analysis and correlation with the VH-subnetwork.

A. Matrix of gene module-trait relationships. Each matrix element contains a correlation value between a gene module's eigenvector and a clinical trait (presence in the VH-subnetwork) and its corresponding p-value and is colored according to the correlation.

B. Module memberships are significantly correlated with gene significance for presence in the VH-subnetwork in "cyan" and "greenyellow" modules.

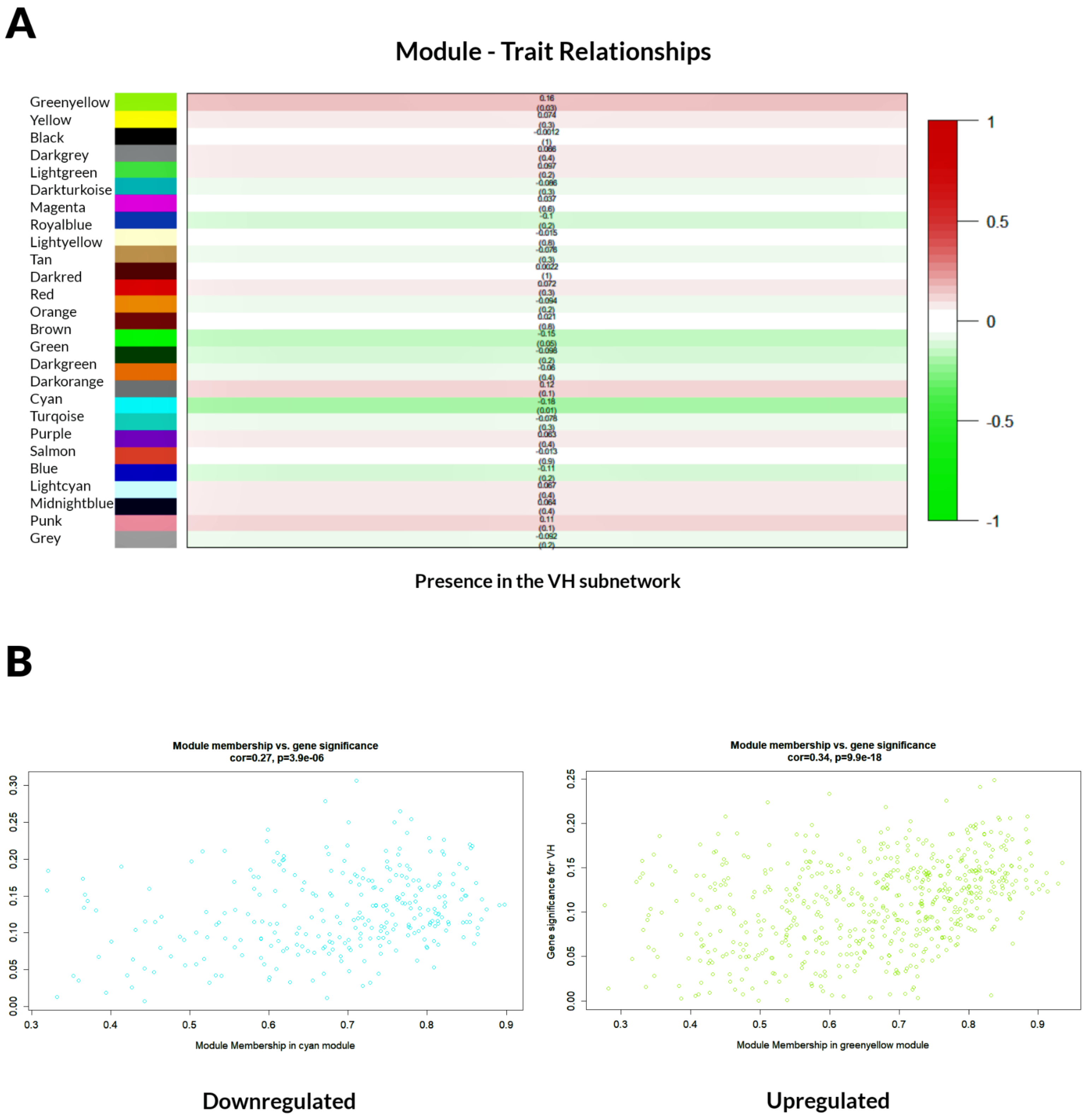

Figure S6: Cell type enrichment of topgenes for downweighted and upweighted VH-associated modules.

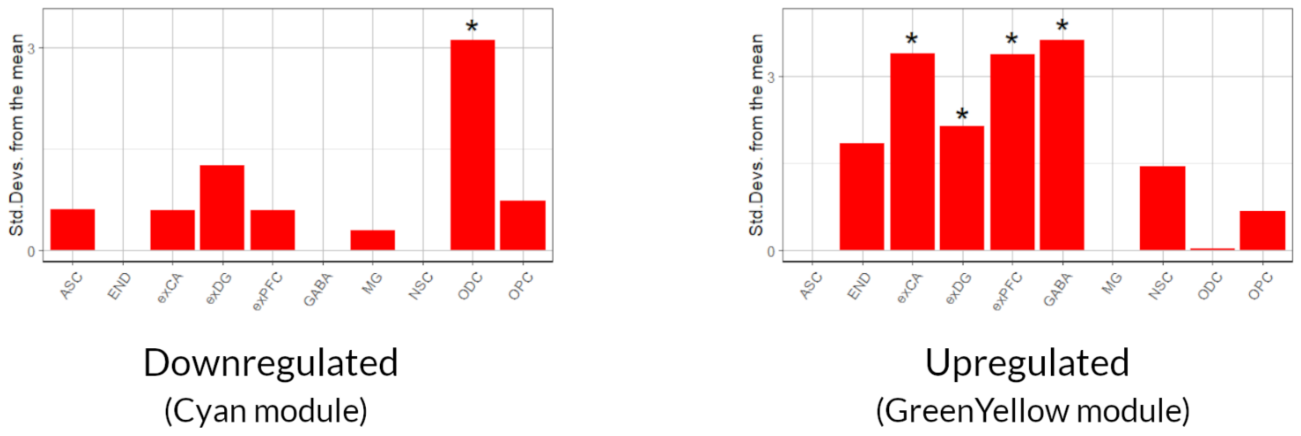

Results presented as standard deviations from the mean; * statistically significant results. ASC: astrocytes, END: endothelial cells, NSC: neuronal stem cells, OPC: Oligodendrocyte Precursor cells, MC: Microglia, exCA: Pyramidal CA, exPFC: Pyramidal prefrontal cortex, exDG: Granule neurons, hippocampus dentate gyrus, GABA: GABAergic interneurons, ODC: Oligodendrocytes.

Additional Files

Filename	Description
**TableS5_GeneListNetworks.xlsx**	**Table S5**. List of genes, module membership and gene significance values for the genes in the cyan and greenyellow modules (VH-associated modules).
**TableS6_FullGOtermsVHmodules.xlsx**	**Table S6.** List of all significantly enriched GO terms for the genes in the cyan and greenyellow modules (VH-associated modules).

URLs

Allen Human Brain Atlas: http://human.brain-map.org/static/download; AIBS single cell data: https://portal.brain-map.org/atlases-and-data/rnaseq; EWCE: https://github.com/NathanSkene/EWCE; g:Profiler: https://biit.cs.ut.ee/gprofiler/gost; REVIGO: http://revigo.irb.hr.

References

Bernhardt BC, Fadaie F, Liu M, Caldairou B, Gu S, Jefferies E, et al. Temporal lobe epilepsy: Hippocampal pathology modulates connectome topology and controllability. Neurology 2019; 92: e2209–e2220.

Gu S, Betzel RF, Mattar MG, Cieslak M, Delio PR, Grafton ST, et al. Optimal trajectories of brain state transitions. NeuroImage 2017; 148: 305–317.

Gu S, Pasqualetti F, Cieslak M, Telesford QK, Yu AB, Kahn AE, et al. Controllability of structural brain networks. Nat Commun 2015; 6: 1–10.

Papapetropoulos S, Katzen H, Schrag A, Singer C, Scanlon BK, Nation D, et al. A questionnaire-based (UM-PDHQ) study of hallucinations in Parkinson's disease. BMC Neurology 2008; 8: 21.
